# Unitary Coupled Cluster: Seizing the Quantum Moment

**DOI:** 10.1021/acs.jpca.3c02781

**Published:** 2023-07-31

**Authors:** Ilias Magoulas, Francesco A. Evangelista

**Affiliations:** Department of Chemistry and Cherry Emerson Center for Scientific Computation, Emory University, Atlanta, Georgia 30322, United States

## Abstract

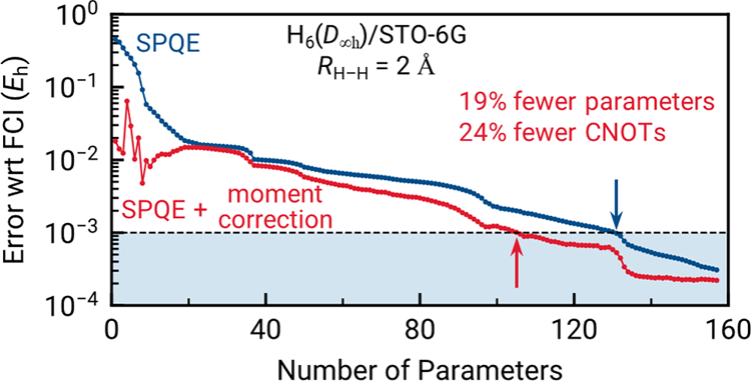

Shallow, CNOT-efficient quantum circuits are crucial for performing accurate computational chemistry simulations on current noisy quantum hardware. Here, we explore the usefulness of noniterative energy corrections, based on the method of moments of coupled-cluster theory, for accelerating convergence toward full configuration interaction. Our preliminary numerical results relying on iteratively constructed ansätze suggest that chemically accurate energies can be obtained with substantially more compact circuits, implying enhanced resilience to gate and decoherence noise.

The first electronic structure calculation on quantum hardware was reported more than 10 years ago.^1^ It involved the iterative quantum phase estimation computation of the spectrum of H_2_ in a minimum basis set. Since then, in tandem with various technological innovations, significant algorithmic advances have taken place aimed at the full utilization of the current generation of noisy intermediate-scale quantum devices.^2^ Of particular importance is the arsenal of hybrid quantum–classical approaches,^3,4^ including the variational (VQE),^5−9^ contracted,^10^ and projective^11^ (PQE) quantum eigensolvers, quantum imaginary time evolution,^12,13^ and quantum subspace diagonalization techniques,^12,14−17^ to mention a few. Such schemes require much shallower circuits than pure quantum algorithms, e.g., quantum phase estimation,^18−20^ substantially reducing the computational cost and sensitivity to gate and decoherence noise.

To pave the way toward practical and robust computations on actual quantum devices, various schemes have been devised to reduce the circuit depth of hybrid quantum–classical algorithms even further. In the case of ansatz-dependent approaches, which are of particular importance to this work, the construction of compact, yet sufficiently expressive, trial states is crucial. In general, the trial state is expressed as
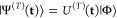
1where **t** = (*t*_1_, *t*_2_, ···) is a vector of parameters, |Φ⟩ is a reference state that can be easily realized on the quantum device, and
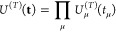
2is the unitary operator that rotates |Φ⟩ to the trial state |Ψ^(*T*)^(**t**)⟩. Equation 2 immediately reveals two complementary strategies for reducing the circuit complexity of ansatz-dependent algorithms. First, it is beneficial to minimize the number of parameters entering *U*^(*T*)^, without, however, significantly sacrificing the expressivity of the ansatz. It is, thus, not surprising that fixed ansätze have been almost invariably replaced by their iteratively constructed counterparts.^10,11,21−27^ In doing so, one eliminates the typically large number of superfluous parameters inadvertently incorporated in fixed-ansätze approaches. Second, the form of the individual unitaries *U*_μ_^(*T*)^ should be such that the corresponding quantum circuits are as compact as possible. To that end, different flavors of ansätze have been explored, typically derived from the unitary extension^28−40^ of coupled-cluster theory^41−46^ (UCC). In this case, the *U*_μ_^(*T*)^(*t*_μ_) unitaries have the form
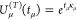
3where κ_μ_ is a generic fermionic anti-Hermitian operator. In the language of second quantization and in the case of an *n*-body operator, κ_μ_ is defined as
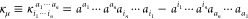
4where *a*_*p*_ (*a*^*p*^ ≡ *a*_*p*_^†^) is the annihilation (creation) operator acting on spinorbital ϕ_*p*_ and indices *i*_1_, *i*_2_, ... or *i*, *j*, ... (*a*_1_, *a*_2_, ... or *a*, *b*, ...) label spinorbitals occupied (unoccupied) in |Φ⟩. Although starting from a fermionic UCC unitary and translating it to the qubit space is a natural choice for the study of electronic structure problems, it generally leads to circuits containing a substantial number of CNOT gates. This is particularly problematic, since current hardware implementations of two-qubit gates, such as CNOTs, are typically ∼10 times noisier than their one-qubit analogs. More CNOT-efficient ansätze are obtained, for example, by directly building UCC in the qubit space^22,24,47^ or employing the fermionic-excitation-based (FEB) circuits and their qubit (QEB) counterparts.^23,25,26,48−50^ Approximate implementations of the FEB and QEB circuits have recently been explored, resulting in single- and two-qubit gate counts that scale linearly with the excitation rank.^51^

Another promising approach for reducing quantum resource requirements that has received relatively little attention is the use of noniterative energy corrections. Such schemes, in conjunction with iteratively constructed ansätze, have the potential to accelerate convergence toward the exact, full configuration interaction (FCI), solution, thus decreasing the depth of the underlying quantum circuits. This avenue was initially explored in the context of the iterative qubit coupled cluster (iQCC) approach^24^ by Ryabinkin et al.^52^ By considering various low-order perturbative corrections to the iQCC energies, they were able to achieve a given level of accuracy with a smaller number of iQCC iterations.

Similar in spirit to the work of Ryabinkin et al., in this study we explore the usefulness of noniterative energy corrections resulting from the formalism of the method of moments of CC (MMCC) equations^53−66^ in accelerating the convergence to FCI of adaptive schemes. Corrections based on the MMCC framework have several desirable properties. Unlike noniterative corrections based on many-body perturbation theory arguments,^67−76^ which generally fail when nondynamical correlations become substantial, MMCC-type corrections are robust in the presence of quasi-degeneracies encountered in typical problems of chemical interest, such as single bond breaking and biradicals. In its exact form, the MMCC framework provides the noniterative correction to the energy of an approximate CC method needed to recover the FCI energy. Although in practice one computes an approximate MMCC correction, improving its quality by incorporating higher-rank moments is straightforward, albeit computationally demanding on a classical machine. Nevertheless, in the case of a unitary ansatz, the necessary ingredients to compute such corrections can be efficiently calculated on a quantum computer (vide infra). Furthermore, UCC-based approaches provide upper bounds to the FCI energy, rendering them immune to the catastrophic failures plaguing traditional single-reference CC methods in the presence of strong correlations, and excellent starting points for noniterative corrections (see also ref (57) for the generalization of the MMCC formalism to extended coupled cluster theory). MMCC methods have been recently introduced to the realm of quantum computing, albeit from a different perspective. Peng and Kowalski have constructed a compact representation of nonunitary operators on quantum devices.^77^ This allowed them to devise quantum algorithms for computing MMCC corrections to conventional, i.e., nonunitary, CC schemes. Although Peng and Kowalski also outline how to extend the MMCC formalism to UCC ansätze in a way similar to our approach, to the best of our knowledge, the corresponding algorithm has never been implemented.

We begin our discussion of noniterative corrections by first summarizing the salient features of the quantum algorithms used to optimize the trial state. We used the adaptive derivative-assembled pseudo-Trotterized VQE (ADAPT-VQE)^21^ and selected PQE (SPQE)^11^ approaches, which are based on iteratively constructed ansätze optimized with VQE and PQE, respectively. Recall that in the VQE and PQE schemes, the energy *E*^(*T*)^ is computed as the expectation value of the Hamiltonian with respect to the trial state,
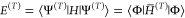
5where ^(*T*)^ = *U*^(*T*)†^*HU*^(*T*)^ is the similarity-transformed Hamiltonian. Thus, both VQE and PQE provide upper bounds to the FCI energy. At a high level, both algorithms rely on essentially the same two alternating steps. First, there is an expansion step promoting the growth of the ansatz. This is accomplished by ordering the operators in a given operator pool based on a predefined importance criterion and adding the most important operator(s) to the ansatz. In the case of VQE, the energy gradients
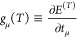
6play the role of the importance criterion, while in PQE the residuals of the UCC equations are used instead. Recall that the UCC residuals are projections onto the manifold of excited Slater determinants (|Φ_μ_⟩ ≡ κ_μ_ |Φ⟩) of the connected cluster form of the Schrödinger equation with the UCC ansatz, eq 1,
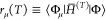
7Note that, as shown in ref (11), the residuals of all operators in the pool can be efficiently estimated by repeated measurements of the “residual state,” defined as
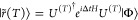
8The second step of the algorithm is the optimization of the ansatz parameters. If we partition the full operator space into those incorporated in the ansatz (*P*) and those excluded (*Q*), then for VQE the optimization is performed variationally, which translates into enforcing the condition *g*_*p*_ = 0 ∀ κ_*p*_ ∈ *P*. In the PQE case, a similar condition is imposed for the residuals (*r*_*p*_ = 0 ∀ κ_*p*_ ∈ *P*). As shown in ref (11), the exact evaluation of a residual element in PQE has the same cost as the exact estimation of the corresponding gradient element in VQE using the parameter shift rule.^78,79^ The remaining details of the SPQE and ADAPT-VQE algorithms can be found in refs (11) and (21), respectively.

Next, we move on with the discussion of the noniterative corrections. Here, we consider two families of noniterative MMCC-type corrections. The first one has the form
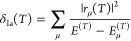
9where *r*_μ_(*T*) are the residuals or moments of the UCC equations^53,54,80^ and *E*_μ_^(*T*)^ = ⟨Φ| |Φ_μ_⟩ the diagonal elements of  Note that the summation over μ excludes the reference determinant. Equation 9 can be regarded as the direct translation of biorthogonal MMCC expansions^59−61^ and their CC(*P*;*Q*) generalization^66,81,82^ to the language of UCC (see also refs^73,83−86^ for earlier noniterative corrections that rely on the left eigenvector of the similarity-transformed Hamiltonian of traditional CC theory). In the numerator of the CC(*P*;*Q*) correction appears the product of moments of CC equations corresponding to the right and left CC states. Recall that traditional CC theory is non-Hermitian and as such the CC similarity-transformed Hamiltonian has distinct right and left eigenvectors. Since the UCC similarity-transformed Hamiltonian is Hermitian, in the translation process, the left-state moment is replaced by the complex conjugate of the right moment. From an alternative point of view, eq 9 can be derived using Löwdin’s partitioning procedure taking the zeroth-order part of the Hamiltonian to be the diagonal elements of (87)

In our current implementation, the summation appearing in eq 9 is performed over all excited Slater determinants |Φ_μ_⟩ for which *r*_μ_(*T*) ≠ 0, namely, |Φ_μ_⟩ ∈ *Q* for PQE and |Φ_μ_⟩ ∈ *P* ⊕*Q* in the case of VQE. For example, in a UCCSD calculation relying on PQE optimization (UCCSD-PQE), the optimum parameters are obtained by imposing the residual conditions
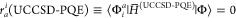
10and
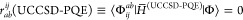
11Thus, the summation in eq 9 defining the δ_Ia_ (UCCSD-PQE) moment correction runs over all triply, quadruply, etc. excited Slater determinants (note that, unlike the case of traditional CCSD where the moment expansion naturally terminates after hextuples, for UCCSD-PQE all moments corresponding to higher-than-double excitations are, in principle, nonzero). Alternatively, in a VQE-based UCCSD (UCCSD-VQE) computation, the parameter optimization is performed variationally, i.e., by enforcing
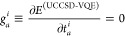
12and
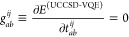
13Consequently, since no residual condition is imposed, the δ_Ia_(UCCSD-VQE) noniterative correction involves all UCCSD-VQE moments, including those corresponding to singles and doubles. In this case, the appropriate schemes are based on the generalized MMCC formalism that was introduced in the context of extended coupled cluster theory.^57^

Next, we consider the computational burden associated with the evaluation of eq 9. To this end, we examine the availability of its constituents. In the case of SPQE, the *r*_μ_(SPQE) residuals are already available, since they are generated for the selection of new operators. This is analogous to how perturbative energy corrections are evaluated in selected configuration interaction (CI) techniques, such as the CI method using perturbative selection made iteratively,^88−90^ adaptive CI,^91,92^ and adaptive sampling CI,^93^ to mention a few. In the case of ADAPT-VQE simulations, the residuals need to be evaluated, but this can be efficiently accomplished by repeated measurements of the residual vector, as discussed above. Furthermore, both SPQE and ADAPT-VQE have access to the *E*^(*T*)^ energy of the trial state, as part of the parameter optimization process. Thus, the major computational overhead is the evaluation of the diagonal elements of the similarity-transformed Hamiltonian in every macro-iteration. To reduce the computational cost associated with the evaluation of *E*_μ_^(*T*)^, we also consider two approximations to the denominators that enter into eq 9. In the first one, denoted as δ_Ib_(*T*), we replace the similarity-transformed Hamiltonian in *E*_μ_^(*T*)^ by the bare Hamiltonian,
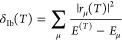
14This allows us to efficiently evaluate the *E*_μ_ = ⟨Φ_μ_|*H*|Φ_μ_⟩ energies only once per simulation using a classical algorithm. The second approximation, designated as δ_Ic_(*T*), is more drastic, replacing the entire denominator appearing in eq 9 by its Møller–Plesset counterpart,
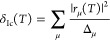
15where Δ_μ_ ≡Δ_*i*_1_ ··· *i*_*n*__^*a*_1_ ··· *a*_*n*_^ = ϵ_*i*_1__ + ···+ ϵ_*i*_*n*__ – ϵ_*a*_1__ – ϵ_*a*_*n*__, with ϵ_*p*_ denoting the Hartree–Fock energy of spinorbital ϕ_*p*_. Note that unlike eqs 9 and 15, the approximation defined in eq 14 is not rigorously size consistent, i.e., it is not additively separable for noninteracting fragments (see the Supporting Information for the proof and underlying conditions for the size consistency of eqs 9 and 15). Nevertheless, our preliminary numerical results suggest that this is not a major issue (vide infra). It is worth mentioning that the noniterative corrections given by eqs 9, 14, and 15 are size-extensive since they involve only connected diagrams.

The second family of noniterative corrections we consider is based on the one defining the original renormalized and completely renormalized CC approaches,^53−55^ namely,
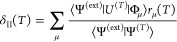
16In eq 16, |Ψ^(ext)^⟩ is the wavefunction from an external source and serves as an approximation to the FCI wavefunction. If |Ψ^(ext)^⟩ ≡ |Ψ^(FCI)^⟩, then eq 16 yields the exact MMCC correction, i.e., *E*^(*T*)^ + δ_II_(*T*) = *E*^(FCI)^. In this regard, eq 16 shares the same philosophy with externally corrected CC approaches.^94−106^ The derivation of this noniterative correction can be found in the Appendix of Peng and Kowalski.^77^ As was the case with eqs 9, 14, and 15, the summation appearing in eq 16 involves all excited Slater determinants |Φ_μ_⟩ for which *r*_μ_(*T*) ≠ 0. An intriguing aspect of this noniterative correction is its flexibility regarding the external state. One has the possibility of utilizing any quantum algorithm that can produce an approximate eigenstate of the Hamiltonian and combining it with any ansatz-dependent scheme with the intention of improving the results of both.

Before we proceed with the discussion of our numerical simulations, we would like to comment on the orbital invariance of the moment energy corrections introduced in our work. The use of Epstein–Nesbet-like denominators in eqs 9 and 14 signifies the lack of invariance under rotations among degenerate orbitals. However, the issue goes deeper. Unlike the full UCC approach, Trotterized UCC unitaries, eq 2, are not orbital invariant. Consequently, none of the noniterative corrections examined in our work possess this formal property by construction. Nevertheless, preliminary numerical results indicate that the loss of orbital invariance in the UCC case is not significant.

To assess the ability of the noniterative, MMCC-type, energy corrections explored in this study to accelerate convergence toward FCI and reduce the resources used by the underlying quantum algorithms, we performed numerical simulations of the symmetric dissociation of the H_6_ linear chain, as described by the minimum STO-6G basis.^107^ In these preliminary calculations, we considered three representative points along the potential energy curve, characterized by the distances between neighboring H atoms of *R*_H–H_ = 1.0, 2.0, and 3.0 Å, corresponding to the equilibrium, recoupling, and strongly correlated regions of the potential, respectively. The underlying quantum algorithms used to gauge the performance of the δ_I_(*T*) corrections, eqs 9, 14, and 15, were SPQE and ADAPT-VQE. To further reduce the depth of the resulting circuits, we used the CNOT-efficient FEB-SPQE (fermionic operators) and QEB-ADAPT-VQE (qubit operators) variants. In the case of SPQE, we also considered the approximate FEB scheme (aFEB), which has been shown to faithfully reproduce the parent FEB-SPQE energetics with negligible symmetry breaking and a linear CNOT count.^51^ In benchmarking the δ_II_(*T*) correction, the trial state was obtained with VQE using a UCCSD ansatz. As a proof of principle, the external states were initially taken to be UCCGSD and UCCSDTQPH, optimized with VQE and PQE, respectively. Both UCCGSD and UCCSDTQPH are exact in the case of H_6_/STO-6G, and the δ_II_(UCCSD) correction to the UCCSD energies reproduced the FCI result with microhartree or better accuracy. In the production run, the external states were those extracted at every macro-iteration of QEB-ADAPT-VQE simulations. This approach shares the same philosophy with the externally corrected CCSD methods based on FCI quantum Monte Carlo^104^ and selected CI wavefunctions^105,106^ (see also the semi-stochastic CC(*P*;*Q*) scheme based on FCI quantum Monte Carlo^108^ and its selected CI counterpart^109^).

All SPQE simulations used a full operator pool and macro-iteration threshold of 10^–2^*E*_h_. The PQE micro-iteration threshold was set to 10^–5^*E*_h_, while the direct inversion of the iterative subspace^110−112^ was utilized to accelerate convergence. Although in typical SPQE runs, multiple operators are added to the ansatz per macro-iteration, here we followed the ADAPT-VQE paradigm and added operators sequentially. This allowed us to properly examine the ability of the noniterative corrections to accelerate convergence of SPQE toward FCI. The ADAPT-VQE simulations relied on a pool of generalized singles and doubles^113,114^ and macro- and micro-iteration thresholds of 10^–3^*E*_h_ and 10^–5^*E*_h_, respectively. All numerical simulations reported in this work have been performed using QForte,^115^ a state-vector emulator developed by our group. The noniterative energy corrections explored in this study have been implemented in a local version of QForte. All computations used restricted Hartree–Fock references, with the one- and two-electron integrals obtained from Psi4.^116^

We begin the discussion of our numerical results by examining the ability of the noniterative, MMCC-type, energy corrections defined in eqs 9, 14, and 15 to accelerate the convergence of FEB-SPQE simulations toward FCI. To that end, in the top panels of Figure 1 we compare the energies resulting from FEB-SPQE and FEB-SPQE_I*x*_ ≡ FEB-SPQE+δ_I*x*_, with *x* = a, b, c. A quick inspection of Figure 1 immediately reveals that all three corrections are capable of reproducing the FCI data within the chemical accuracy of 1 m*E*_h_ more rapidly than the underlying FEB-SPQE approach. Their performance is particularly impressive in the weakly and moderately correlated regions, requiring as much as ∼45 and ∼20% fewer parameters than FEB-SPQE. This emphasizes the exceptional ability of such noniterative corrections to recover dynamical correlation effects. The decrease in the number of parameters is accompanied by similarly remarkable reductions in the CNOT counts of the corresponding ansatz circuits, as illustrated in the bottom panels of Figure 1. In comparing the various noniterative corrections among themselves, the FEB-SPQE_Ic_ scheme that relies on the standard Møller–Plesset denominator has the least favorable performance. With the exception of the very early stages of the FEB-SPQE simulations, FEB-SPQE_Ib_ faithfully reproduces the results of its FEB-SPQE_Ia_ parent. This remains true even near the dissociation threshold of H_6_, represented by the geometry characterized by *R*_H–H_ = 3.0 Å. This implies that the size inconsistency introduced in the denominator of eq 14 is not severe, at least in the case of H_6_. As a result, in the subsequent benchmarks, we will be focusing on the δ_Ib_ correction, since it not only provides results similar to the more complete δ_Ia_ scheme, but is also more computationally efficient (vide supra).

**Figure 1:**
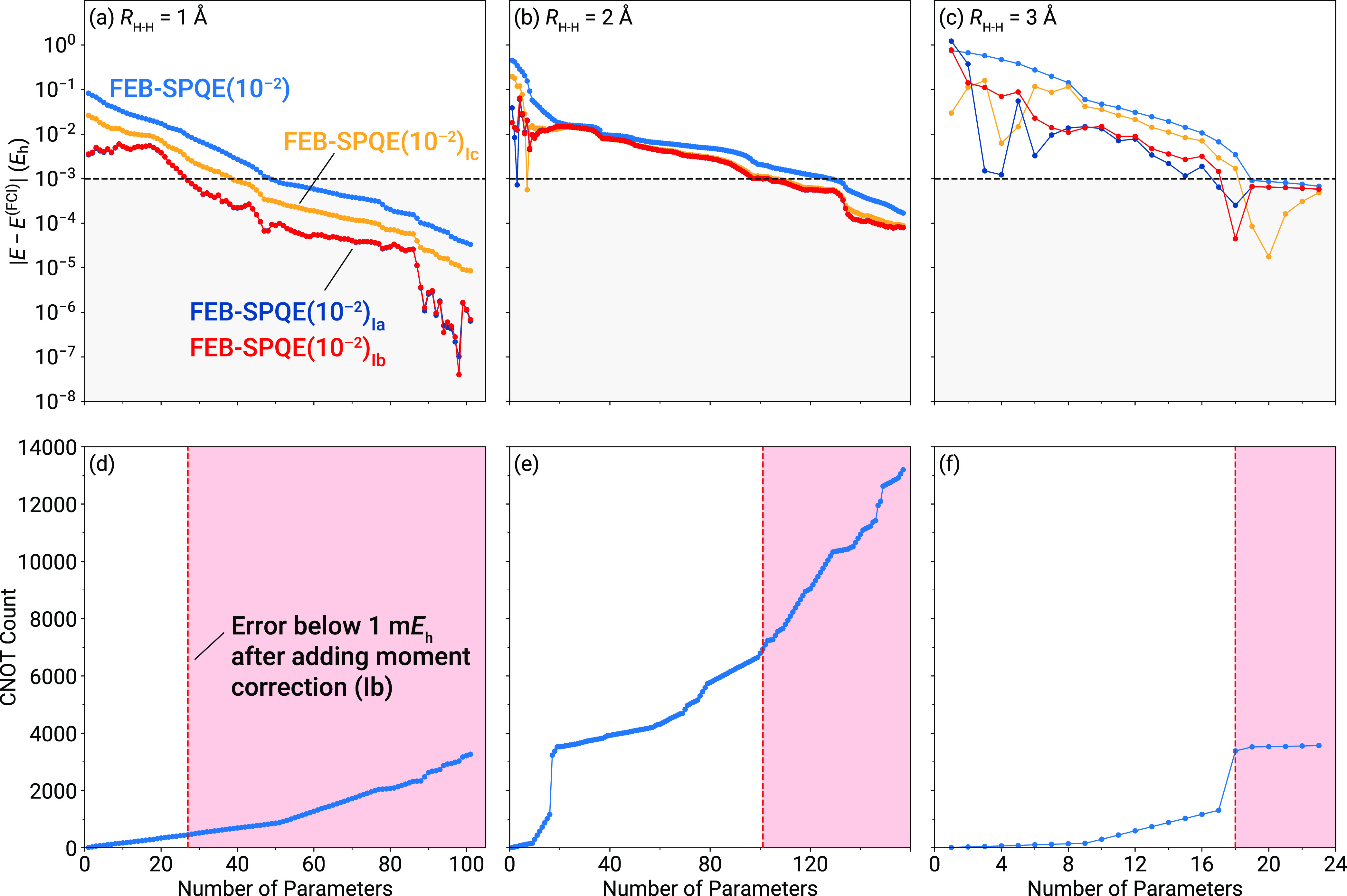
Errors relative to FCI [(a)–(c)] and CNOT gate counts [(d)–(f)] characterizing the FEB-SPQE simulations of the symmetric dissociation of the H_6_/STO-6G linear chain at three representative distances between neighboring H atoms, including *R*_H–H_ = 1.0 Å [(a) and (d)], *R*_H–H_ = 2.0 Å [(b) and (e)], and *R*_H–H_ = 3.0 Å [(c) and (f)]. The gray-shaded area in the top-row panels indicates results within chemical accuracy (1 m*E*_h_) from FCI. The red-shaded area in the bottom-row panels denotes the CNOT counts of the underlying FEB-SPQE quantum circuits for which the FEB-SPQE_Ib_ energies are within chemical accuracy.

In an effort to reduce the circuit depth even further, we combine the δ_Ib_ noniterative correction with aFEB-SPQE. As already mentioned above, the recently introduced aFEB circuits are approximate implementations of the CNOT-efficient FEB ones that require a number of CNOT gates that scales linearly with the excitation rank of a given operator.^51^ Furthermore, although the aFEB approximation breaks the particle number *N* and total spin projection *S*_*z*_ symmetries, it has been demonstrated that aFEB-SPQE is characterized by an essentially negligible symmetry contamination and a faithful reproduction of the parent FEB-SPQE energetics. As might have been anticipated, the same is true when we examine the energies resulting from their δ_Ib_-corrected counterparts. Indeed, as shown in Figure S1 of the Supporting Information, the aFEB-SPQE_Ib_ data are practically indistinguishable from those obtained with FEB-SPQE_Ib_. This is even true in the recoupling region, characterized by *R*_H–H_ = 2.0 Å, where the largest deviation, on the order of 0.1 m*E*_h_, is observed. At this point, it is worth mentioning that due to the symmetry breaking introduced by the aFEB quantum circuits, aFEB-SPQE has nonzero moments corresponding to Slater determinants with *N* ≠ 6 and/or *S*_*z*_ ≠ 0 for the ground electronic state of H_6_. In principle, one could incorporate such Fock-space moments into the MMCC-type noniterative corrections to improve the quality of the results even further. However, since the contribution of the symmetry contaminants to the aFEB-SPQE wavefunction is negligible,^51^ we did not consider such a generalized correction.

Next, we examine the usefulness of the δ_Ib_ noniterative correction in the context of ADAPT-VQE (QEB-ADAPT-VQE_Ib_) and compare the results with those of aFEB-SPQE_Ib_. When examining the errors with respect to FCI, we observe that, in general, the QEB-ADAPT-VQE_Ib_ approach offers a major improvement over the underlying QEB-ADAPT-VQE energetics (top panels of Figure 2). This enables QEB-ADAPT-VQE_Ib_ to recover the FCI data within 1.0 m*E*_h_ faster than the underlying QEB-ADAPT-VQE approach, resulting in substantial savings in terms of CNOTs (see the bottom panels of Figure 2). Exception to this is the QEB-ADAPT-VQE simulation for the strongly correlated regime of H_6_. In this case, QEB-ADAPT-VQE is characterized by a very slow convergence to FCI, most likely due to the presence of a local energy minimum. For QEB-ADAPT-VQE_Ib_, this manifests itself as a plateau in the corresponding energies. The fact that after about 120 parameters the QEB-ADAPT-VQE and QEB-ADAPT-VQE_Ib_ results practically coincide, i.e., the QEB-ADAPT-VQE moments are close to zero, further corroborates to the fact that the QEB-ADAPT-VQE wavefunction closely resembles that of an exact excited electronic state. Eventually, both QEB-ADAPT-VQE and QEB-ADAPT-VQE_Ib_ achieve chemical accuracy at the same time, requiring about 80% of the number of parameters of FCI.

**Figure 2:**
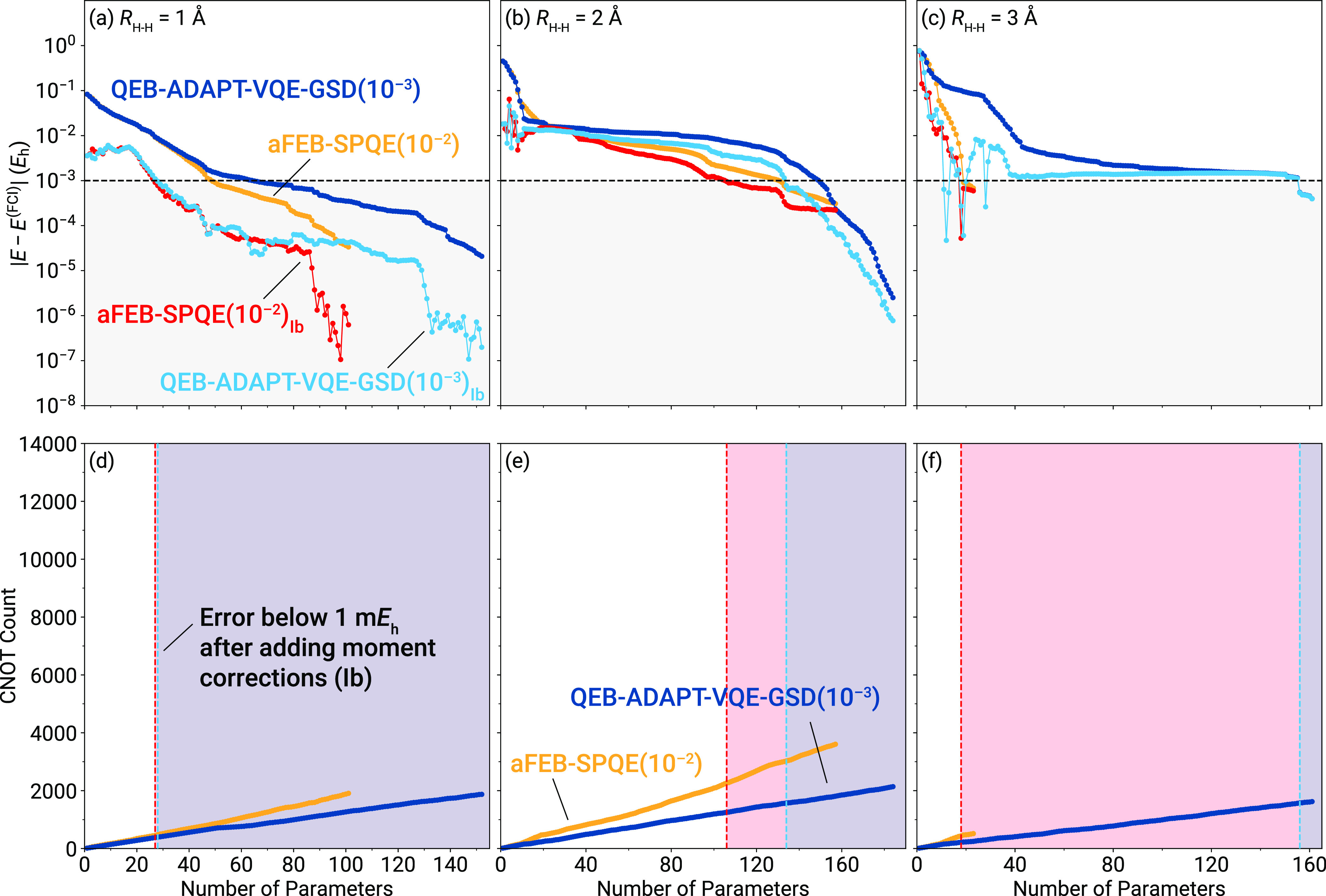
Errors relative to FCI [(a)–(c)] and CNOT gate counts [(d)–(f)] characterizing the aFEB-SPQE and QEB-ADAPT-VQE-GSD simulations of the symmetric dissociation of the H_6_/STO-6G linear chain at three representative distances between neighboring H atoms, including *R*_H–H_ = 1.0 Å [(a) and (d)], *R*_H–H_ = 2.0 Å [(b) and (e)], and *R*_H–H_ = 3.0 Å [(c) and (f)]. The gray-shaded area in the top-row panels indicates results within chemical accuracy (1 m*E*_h_) from FCI. The blue- and red-shaded areas in the bottom-row panels denote the CNOT counts of the underlying aFEB-SPQE and QEB-ADAPT-VQE-GSD quantum circuits, respectively, for which the aFEB-SPQE_Ib_ and QEB-ADAPT-VQE-GSD_Ib_ energetics are within chemical accuracy.

Moving on to the comparison of the ADAPT-VQE and SPQE approaches, a quick inspection of the top panels of Figure 2 immediately reveals that aFEB-SPQE provides, in general, more accurate energetics than QEB-ADAPT-VQE for the same number of parameters. Similar behavior is observed when we examine their MMCC-corrected counterparts. The fact that aFEB-SPQE_Ib_ is characterized by a faster convergence to FCI than QEB-ADAPT-VQE_Ib_ does not necessarily guarantee that it does so with fewer number of CNOTs. As demonstrated in the bottom panels of Figure 2, although both aFEB-SPQE and QEB-ADAPT-VQE generate quantum circuits with CNOT counts that scale, more or less, linearly with the number of parameters, the prefactor is smaller in the case of QEB-ADAPT-VQE. Indeed, for the three examined geometries of H_6_, characterized by the *R*_H–H_ values of 1.0, 2.0, and 3.0 Å, QEB-ADAPT-VQE_Ib_ attains chemical accuracy with the same, less, and more CNOTs, respectively, than when aFEB-SPQE_Ib_ does.

Now we turn our attention to the δ_II_ MMCC-type correction, given by eq 16. To gauge the performance of this correction, we considered the UCCSD_II_ scheme in which we corrected the VQE UCCSD energies using the wavefunctions resulting from each macro-iteration defining the QEB-ADAPT-VQE simulations. The reason that we opted to use QEB-ADAPT-VQE as the external source of the wavefunction rather than aFEB-SPQE is that, for the same number of parameters, the former generates the most CNOT-efficient circuits. In Figure 3, we present the errors relative to FCI characterizing the UCCSD_II_ method and the underlying QEB-ADAPT-VQE approach. As depicted in Figure 3, with the exception of the very early stages of the QEB-ADAPT-VQE simulations, in which UCCSD is more accurate, UCCSD_II_ consistently outperforms both UCCSD and QEB-ADAPT-VQE. As far as the number of CNOTs needed to achieve chemical accuracy is concerned, which of the UCCSD and QEB-ADAPT-VQE requires the deepest circuit depends on the strength of the correlation effects. Based on our numerical results for H_6_, in the weakly and strongly correlated regions, where UCCSD_II_ recovers the FCI energies within 1.0 m*E*_h_ rather quickly, UCCSD is the bottleneck in terms of CNOT count. The situation changes when we examine the recoupling region, in which case QEB-ADAPT-VQE defines the overall CNOT cost. In comparing the UCCSD_II_ and QEB-ADAPT-VQE_Ib_ approaches among themselves, we observe that UCCSD_II_ is characterized by a more rapid convergence to FCI. This can be mostly attributed to the fact that UCCSD offers a good starting point for the MMCC-type correction, especially so during the early stages of the simulation.

**Figure 3:**
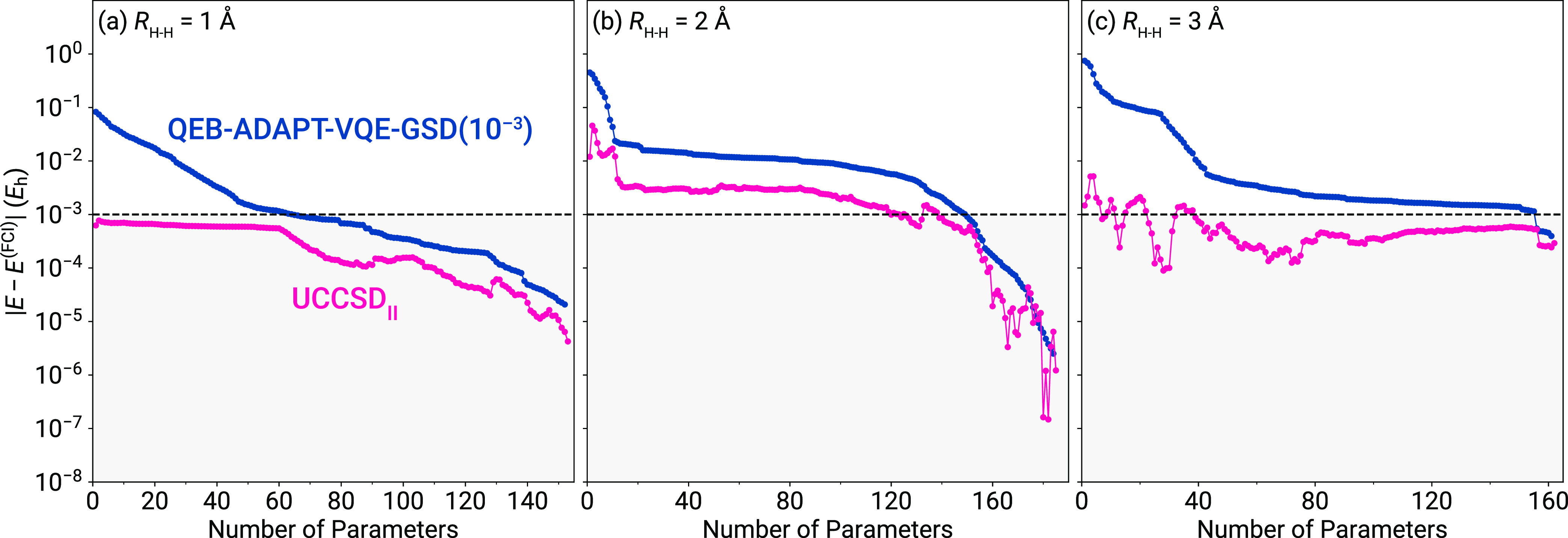
Errors relative to FCI characterizing the moment-corrected UCCSD_II_ scheme and the underlying QEB-ADAPT-VQE-GSD simulations of the symmetric dissociation of the H_6_/STO-6G linear chain at three representative distances between neighboring H atoms, including (a) *R*_H–H_ = 1.0 Å, (b) *R*_H–H_ = 2.0 Å, and (c) *R*_H–H_ = 3.0 Å. The gray-shaded area indicates results within chemical accuracy (1 m*E*_h_) from FCI.

In this work, we explored the usefulness of moment expansions, used in the past to define the renormalized and completely renormalized CC approaches, to construct noniterative corrections to the energies obtained with hybrid quantum–classical algorithms employing a UCC ansatz. In an attempt to minimize the circuit depth, we combined the noniterative energy corrections with iteratively constructed ansätze and CNOT-efficient implementations of quantum circuits. Our preliminary numerical results for three representative geometries along the potential energy curve characterizing the symmetric dissociation of the H_6_ linear chain are very encouraging. All of the examined corrections accelerated the convergence to FCI of the underlying quantum algorithms, resulting in substantial savings in terms of CNOT gates. The performance of the aFEB-SPQE_Ib_, QEB-ADAPT-VQE_Ib_, and UCCSD_II_ is particularly promising. We point out that the corrections studied in this work are applicable even when the trial state is not of the UCC form, opening the possibility to improve hardware-efficient ansätze and other trial states that are not expressible via the UCC form. In the future, we will explore the flexibility of the δ_II_ correction, combining different types of trial and external wavefunctions.

Another aspect worth examining is the capability of our noniterative corrections to enable simulations using larger-than-minimum basis sets. Taking the example of the H_6_/cc-pVDZ system, one can perform a VQE/PQE simulation on a minimal, valence active space to capture static correlations. Subsequently, a moment energy correction, based on determinants outside of the active space, can be used to estimate dynamical correlations. A similar approach has already been proposed in the context of perturbative energy corrections for iQCC.^52^

Finally, inspired by the success of MMCC-type corrections for excited electronic states,^61,63,66,117−122^ we plan to investigate this aspect as well.

## Supporting Information Available

Proof of the size consistency of the noniterative corrections and additional numerical results (PDF)
Numerical data (XLXS)
