# Supplementary material for: Unitary Coupled Cluster: Seizing the Quantum Moment
Source: J Phys Chem A. 2023 Jul 31;127(31):6567–76. doi: 10.1021/acs.jpca.3c02781 (PMC10424243; doi:10.1021/acs.jpca.3c02781)
Supplement: Supplementary file 1 — jp3c02781_si_001.pdf [file jp3c02781_si_001.pdf]

Supporting Information: Unitary Coupled Cluster: Seizing the Quantum Moment

Ilias Magoulas* and Francesco A. Evangelista*

Department of Chemistry and Cherry Emerson Center for Scientific Computation, Emory University, Atlanta, Georgia 30322, USA

E-mail: ilias.magoulas@emory.edu; francesco.evangelista@emory.edu

This Supporting Information document is organized as follows. In Section S1 we provide the proof and underlying conditions for the size-consistency of the non-iterative energy corrections, derived from the formalism of the method of moments of coupled-cluster (MMCC) equations, considered in the main text. Section S2 contains, in a graphical form, additional numerical results.

The numerical data generated in this study can be found in the Excel file that forms part of the present Supporting Information.

S1 Method of Moments of Coupled-Cluster Equations and Unitary Coupled Cluster: Size Consistency

Here, we provide a mathematical proof of the size-consistency of the MMUCC formulas considered in the main text, along with the associated necessary and sufficient conditions.

We begin our analysis by examining the isolated systems A and B, characterized by the H_A and H_B Hamiltonians, respectively. The Schrödinger equations for the ground electronic states of each of the two systems read

$$H_A \left|\Psi_{0,A}\right\rangle = E_{0,A} \left|\Psi_{0,A}\right\rangle \tag{S1}$$

and

$$H_B \left|\Psi_{0,B}\right\rangle = E_{0,B} \left|\Psi_{0,B}\right\rangle . \tag{S2}$$

The exact $\left|\Psi_{0,A}\right\rangle$ and $\left|\Psi_{0,B}\right\rangle$ electronic states can be expressed in terms of the following unitary parametrizations:

$$\left|\Psi_{0,A}\right\rangle = U_A \left|\Phi_A\right\rangle \tag{S3}$$

and

$$\left|\Psi_{0,B}\right\rangle = U_B \left|\Phi_B\right\rangle , \tag{S4}$$

with U_A and U_B representing products of elementary unitary excitation operators, and $|\Phi_A\rangle$ and $|\Phi_B\rangle$ denoting the reference Slater determinants of systems A and B, respectively. The corresponding sets of all excited determinants afforded by the respective one-electron bases are denoted $\{ |\Phi_{k,A}\rangle\}$ for system A and $\{ |\Phi_{k,B}\rangle\}$ in the case of system B. Without loss of generality, we assume the use of canonical Hartree–Fock orbitals. The many-electron Hilbert space of system A, $\mathcal{H}_A$, can be partitioned as $\mathcal{H}_A = \mathcal{H}_{0,A} \oplus \mathcal{H}_{K,A}$, where $\mathcal{H}_{0,A} = \text{span}(|\Phi_A\rangle)$ and $\mathcal{H}_{K,A} = \text{span}(\{ |\Phi_{k,A}\rangle\})$. Similar definitions apply in the case of system B. The fact that $|\Psi_{0,A}\rangle$ and $|\Psi_{0,B}\rangle$ are exact implies the following residual conditions:

$$\langle\Phi_{k,A}|\bar{H}_A|\Phi_A\rangle = 0, \forall\, |\Phi_{k,A}\rangle \in \mathcal{H}_{K,A} \tag{S5}$$

and

$$\langle\Phi_{k,B}|\bar{H}_B|\Phi_B\rangle = 0, \forall\, |\Phi_{k,B}\rangle \in \mathcal{H}_{K,B}, \tag{S6}$$

where $\bar{H}_A = U_A^\dagger H_A U_A$ and $\bar{H}_B = U_B^\dagger H_B U_B$ are the pertinent similarity-transformed Hamiltonians.

Subsequently, we turn our attention to an approximate description of the isolated systems A and B. In this case, the $U_A^{(P_A)}$ and $U_B^{(P_B)}$ unitaries that define the $|\Psi_{0,A}^{(P_A)}\rangle$ and $|\Psi_{0,B}^{(P_B)}\rangle$ ansätze,

$$|\Psi_{0,A}^{(P_A)}\rangle = U_A^{(P_A)}\,|\Phi_A\rangle \tag{S7}$$

and

$$|\Psi_{0,B}^{(P_B)}\rangle = U_B^{(P_B)}\,|\Phi_B\rangle\,, \tag{S8}$$

contain fewer parameters than the dimensions of the $\mathcal{H}_{K,A}$ and $\mathcal{H}_{K,B}$ spaces, respectively. Consequently, the residual conditions can only be satisfied in the $\mathcal{H}_{P,A} \subset \mathcal{H}_{K,A}$ and $\mathcal{H}_{P,B} \subset \mathcal{H}_{K,B}$ subspaces corresponding to the excitation operators appearing in the $U_A^{(P_A)}$ and $U_B^{(P_B)}$ unitaries:

$$\langle\Phi_{p,A}|\bar{H}_A^{(P_A)}|\Phi_A\rangle = 0, \forall\, |\Phi_{p,A}\rangle \in \mathcal{H}_{P,A} \tag{S9}$$

and

$$\langle\Phi_{p,B}|\bar{H}_B^{(P_B)}|\Phi_B\rangle = 0, \forall\, |\Phi_{p,B}\rangle \in \mathcal{H}_{P,B}. \tag{S10}$$

The $\mathcal{H}_{Q,A} \equiv \mathcal{H}_{K,A} \ominus \mathcal{H}_{P,A}$ and $\mathcal{H}_{Q,B} \equiv \mathcal{H}_{K,B} \ominus \mathcal{H}_{P,B}$ subspaces are spanned by the excited Slater determinants $\{|\Phi_{q,A}\rangle\}$ and $\{|\Phi_{q,B}\rangle\}$, respectively, that do not satisfy the pertinent residual conditions.

Next we consider the electronic structure of the supersystem AB, comprised of the non-interacting systems A and B. Since there is no coupling between the two systems, the Hamiltonian of supersystem AB is

$$H_{AB} = H_A \otimes \mathbf{1}_B + \mathbf{1}_A \otimes H_B \equiv H_A + H_B, \tag{S11}$$

with $\mathbf{1}_A$ and $\mathbf{1}_B$ denoting the identity operators on the $\mathcal{H}_A$ and $\mathcal{H}_B$ many-electron Hilbert spaces, respectively. Due to the fact that factorized UCC ansätze are not orbital-invariant, the choice of orbitals is crucial for achieving size-consistency. To that end, we consider a localized orbital basis, comprised of the canonical Hartree–Fock orbitals of systems A and B. The use of a localized basis and the fact that systems A and B do not interact imply that the unitaries defining the exact and approximate ansätze of supersystem AB will only involve localized excitations, i.e.,

$$|\Psi_{0,AB}\rangle = U_{AB}\,|\Phi_{AB}\rangle = U_A U_B\,|\Phi_{AB}\rangle \tag{S12}$$

and

$$|\Psi_{0,AB}^{(P_A,P_B)}\rangle = U_{AB}^{(P_A,P_B)}\,|\Phi_{AB}\rangle = U_A^{(P_A)} U_B^{(P_B)}\,|\Phi_{AB}\rangle\,, \tag{S13}$$

with $|\Phi_{AB}\rangle$ being the reference determinant of the supersystem AB. Furthermore, the operators H_A, U_A, and $U_A^{(P_A)}$ and H_B, U_B, and $U_B^{(P_B)}$ pairwise commute since they are acting on different spaces. Note that to ensure the proper multiplicative separability of the wavefunction and additivity of the energy, the ordering of the elementary anti-Hermitian excitation

operators defining the U_A, U_B, $U_A^{(P_A)}$, and $U_B^{(P_B)}$ unitaries needs to be preserved between the computations for the isolated systems A and B and the non-interacting supersystem AB. Assuming that $|\Phi_{AB}\rangle$ is separable, namely,

$$|\Phi_{AB}\rangle = |\Phi_A\rangle \otimes |\Phi_B\rangle \equiv |\Phi_A\rangle\, |\Phi_B\rangle\,, \tag{S14}$$

we arrive at the separability of the underlying $|\Psi_{0,AB}\rangle$ and $|\Psi_{0,AB}^{(P_A,P_B)}\rangle$ states:

$$|\Psi_{0,AB}\rangle = U_A\,|\Phi_A\rangle\, U_B\,|\Phi_B\rangle = |\Psi_{0,A}\rangle\, |\Psi_{0,B}\rangle \tag{S15}$$

and

$$|\Psi_{0,AB}^{(P_A,P_B)}\rangle = U_A^{(P_A)}\,|\Phi_A\rangle\, U_B^{(P_B)}\,|\Phi_B\rangle = |\Psi_{0,A}^{(P_A)}\rangle\, |\Psi_{0,B}^{(P_B)}\rangle\,. \tag{S16}$$

First, we consider the δ_{II} MMCC correction, eq (16) of the main text, since the proof is somewhat more involved. It is straightforward to show that in the case of the non-interacting supersystem AB, $\delta_{\mathrm{II},0}^{(P_A,P_B)}$ becomes

$$\begin{aligned}\delta_{\mathrm{II},0}^{(P_A,P_B)} = & \sum_{|\Phi_{q,A}\rangle \in \mathcal{H}_{Q,A}} \frac{\langle\Psi_{0,AB}|U_{AB}^{(P_A,P_B)}|\Phi_{q,A}\rangle\,|\Phi_B\rangle\,\langle\Phi_{q,A}|\,\langle\Phi_B|\,\bar{H}_{AB}^{(P_A,P_B)}\,|\Phi_A\rangle\,|\Phi_B\rangle}{\langle\Psi_{0,AB}|\Psi_{0,AB}^{(P_A,P_B)}\rangle} + \\ & \sum_{|\Phi_{q,B}\rangle \in \mathcal{H}_{Q,B}} \frac{\langle\Psi_{0,AB}|U_{AB}^{(P_A,P_B)}|\Phi_A\rangle\,|\Phi_{q,B}\rangle\,\langle\Phi_A|\,\langle\Phi_{q,B}|\,\bar{H}_{AB}^{(P_A,P_B)}\,|\Phi_A\rangle\,|\Phi_B\rangle}{\langle\Psi_{0,AB}|\Psi_{0,AB}^{(P_A,P_B)}\rangle} + \\ & \sum_{\substack{|\Phi_{q,A}\rangle \in \mathcal{H}_{Q,A} \\ |\Phi_{q,B}\rangle \in \mathcal{H}_{Q,B}}} \frac{\langle\Psi_{0,AB}|U_{AB}^{(P_A,P_B)}|\Phi_{q,A}\rangle\,|\Phi_{q,B}\rangle\,\langle\Phi_{q,A}|\,\langle\Phi_{q,B}|\,\bar{H}_{AB}^{(P_A,P_B)}\,|\Phi_A\rangle\,|\Phi_B\rangle}{\langle\Psi_{0,AB}|\Psi_{0,AB}^{(P_A,P_B)}\rangle}\end{aligned}\,. \tag{S17}$$

The above expression can be simplified since

$$\begin{aligned}\langle\Phi_{q,A}|\,\langle\Phi_{q,B}|\,\bar{H}_{AB}^{(P_A,P_B)}\,|\Phi_A\rangle\,|\Phi_B\rangle &= \langle\Phi_{q,A}|\,\langle\Phi_{q,B}|\,(\bar{H}_A^{(P_A)} + \bar{H}_B^{(P_B)})\,|\Phi_A\rangle\,|\Phi_B\rangle \\ &= \langle\Phi_{q,A}|\bar{H}_A^{(P_A)}|\Phi_A\rangle\,\langle\Phi_{q,B}|\Phi_B\rangle + \langle\Phi_{q,B}|\bar{H}_B^{(P_B)}|\Phi_B\rangle\,\langle\Phi_{q,A}|\Phi_A\rangle \\ &= 0,\end{aligned} \tag{S18}$$

where in the last step we used the fact that Slater determinants are orthonormal. Therefore, eq (S17) reduces to

$$\begin{aligned}\delta_{\mathrm{II},0}^{(P_A,P_B)} = & \sum_{|\Phi_{q,A}\rangle \in \mathcal{H}_{Q,A}} \frac{\langle \Psi_{0,AB} | U_{AB}^{(P_A,P_B)} | \Phi_{q,A}\rangle \, |\Phi_B\rangle \, \langle \Phi_{q,A}| \, \langle \Phi_B| \, \bar{H}_{AB}^{(P_A,P_B)} \, |\Phi_A\rangle \, |\Phi_B\rangle}{\langle \Psi_{0,AB} | \Psi_{0,AB}^{(P_A,P_B)}\rangle} + \\ & \sum_{|\Phi_{q,B}\rangle \in \mathcal{H}_{Q,B}} \frac{\langle \Psi_{0,AB} | U_{AB}^{(P_A,P_B)} | \Phi_A\rangle \, |\Phi_{q,B}\rangle \, \langle \Phi_A| \, \langle \Phi_{q,B}| \, \bar{H}_{AB}^{(P_A,P_B)} \, |\Phi_A\rangle \, |\Phi_B\rangle}{\langle \Psi_{0,AB} | \Psi_{0,AB}^{(P_A,P_B)}\rangle}\end{aligned} \, . \tag{S19}$$

Before we are able to proceed any further, we need to evaluate the various quantities appearing in eq (S19). Starting with the denominator that is common in both terms, it is fairly straightforward to show that it factorizes as follows:

$$\langle \Psi_{0,AB} | \Psi_{0,AB}^{(P_A,P_B)}\rangle = \langle \Psi_{0,A} | \Psi_{0,A}^{(P_A)}\rangle \, \langle \Psi_{0,B} | \Psi_{0,B}^{(P_B)}\rangle \, . \tag{S20}$$

Focusing on the numerator of the first term appearing in eq (S19), we have that

$$\begin{aligned}\langle \Psi_{0,AB} | U_{AB}^{(P_A,P_B)} | \Phi_{q,A}\rangle \, |\Phi_B\rangle &= \langle \Phi_A| \, \langle \Phi_B| \, U_A^{\dagger} U_B^{\dagger} U_A^{(P_A)} U_B^{(P_B)} \, |\Phi_{q,A}\rangle \, |\Phi_B\rangle \\ &= \langle \Phi_A | U_A^{\dagger} U_A^{(P_A)} | \Phi_{q,A}\rangle \, \langle \Phi_B | U_B^{\dagger} U_B^{(P_B)} | \Phi_B\rangle \\ &= \langle \Psi_{0,A} | U_A^{(P_A)} | \Phi_{q,A}\rangle \, \langle \Psi_{0,B} | \Psi_{0,B}^{(P_B)}\rangle\end{aligned} \tag{S21}$$

and

$$\begin{aligned}\langle \Phi_{q,A}| \, \langle \Phi_B| \, \bar{H}_{AB}^{(P_A,P_B)} \, |\Phi_A\rangle \, |\Phi_B\rangle &= \langle \Phi_{q,A}| \, \langle \Phi_B| \, (\bar{H_A}^{(P_A)} + \bar{H_B}^{(P_B)}) \, |\Phi_A\rangle \, |\Phi_B\rangle \\ &= \langle \Phi_{q,A} | \bar{H}_A^{(P_A)} | \Phi_A\rangle \, \langle \Phi_B | \Phi_B\rangle + \langle \Phi_B | \bar{H}_B^{(P_B)} | \Phi_B\rangle \, \langle \Phi_{q,A} | \Phi_A\rangle \\ &= \langle \Phi_{q,A} | \bar{H}_A^{(P_A)} | \Phi_A\rangle \, .\end{aligned} \tag{S22}$$

Similar expressions can be derived in the case of the second term appearing in eq (S19).

We are now in a position to derive the final expression for the non-iterative moment

correction for the supersystem. Using the above information, eq (S19) yields

$$
\begin{aligned}
\delta_{\mathrm{II},0}^{(P_A,P_B)} =& \sum_{|\Phi_{q,A}\rangle \in \mathcal{H}_{Q,A}} \frac{\langle \Psi_{0,A}|U_A^{(P_A)}|\Phi_{q,A}\rangle \ \langle \Psi_{0,B}|\Psi_{0,B}^{(P_B)}\rangle \ \langle \Phi_{q,A}|\bar{H}_A^{(P_A)}|\Phi_A\rangle}{\langle \Psi_{0,A}|\Psi_{0,A}^{(P_A)}\rangle \ \langle \Psi_{0,B}|\Psi_{0,B}^{(P_B)}\rangle} + \\
& \sum_{|\Phi_{q,B}\rangle \in \mathcal{H}_{Q,B}} \frac{\langle \Psi_{0,B}|U_B^{(P_B)}|\Phi_{q,B}\rangle \ \langle \Psi_{0,A}|\Psi_{0,A}^{(P_A)}\rangle \ \langle \Phi_{q,B}|\bar{H}_B^{(P_B)}|\Phi_B\rangle}{\langle \Psi_{0,A}|\Psi_{0,A}^{(P_A)}\rangle \ \langle \Psi_{0,B}|\Psi_{0,B}^{(P_B)}\rangle} \\
=& \sum_{|\Phi_{q,A}\rangle \in \mathcal{H}_{Q,A}} \frac{\langle \Psi_{0,A}|U_A^{(P_A)}|\Phi_{q,A}\rangle \ \langle \Phi_{q,A}|\bar{H}_A^{(P_A)}|\Phi_A\rangle}{\langle \Psi_{0,A}|\Psi_{0,A}^{(P_A)}\rangle} + \\
& \sum_{|\Phi_{q,B}\rangle \in \mathcal{H}_{Q,B}} \frac{\langle \Psi_{0,B}|U_B^{(P_B)}|\Phi_{q,B}\rangle \ \langle \Phi_{q,B}|\bar{H}_B^{(P_B)}|\Phi_B\rangle}{\langle \Psi_{0,B}|\Psi_{0,B}^{(P_B)}\rangle} \\
=& \delta_{\mathrm{II},0}^{(P_A)} + \delta_{\mathrm{II},0}^{(P_B)}.
\end{aligned}
\tag{S23}
$$

Therefore, we proved that the MMCC-type correction defined by eq (16) of the main text is size-consistent, provided that the following conditions are satisfied:

1. Use of orbitals localized on the individual fragments.

2. Separability of the underlying reference state.

3. The ordering of the elementary unitary excitation operators needs to be identical in the isolated fragments and the supersystem.

The proof that the δ_{Ia} and δ_{Ic} non-iterative MMCC-type corrections, given by eqs (9) and (15), respectively, of the main text, are also size-consistent follows the same steps as above, depending on the same conditions as well.

S2 Additional Numerical Results

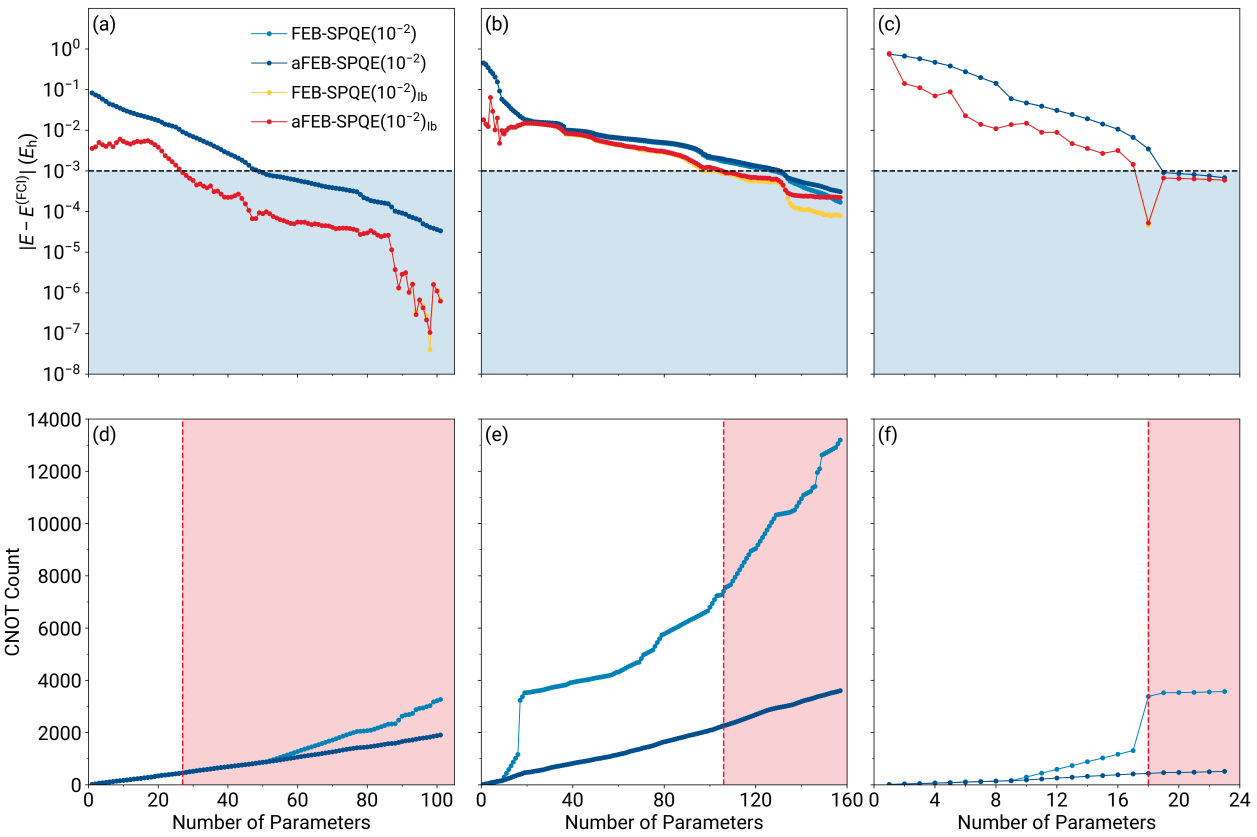

Figure S1: Errors relative to FCI [(a)–(c)] and CNOT gate counts [(d)–(f)] characterizing the aFEB-SPQE simulations of the symmetric dissociation of the H_6/STO-6G linear chain at three representative distances between neighboring H atoms, including $R_{\text{H–H}} = 1.0$ Å [(a) and (d)], $R_{\text{H–H}} = 2.0$ Å [(b) and (e)], and $R_{\text{H–H}} = 3.0$ Å [(c) and (f)]. The blue-shaded area in the top-row panels indicates results within chemical accuracy ($1\,\text{m}E_\text{h}$) from FCI. The red-shaded area in the bottom-row panels denotes the CNOT counts of the underlying aFEB-SPQE quantum circuits for which the aFEB-SPQE$_{\text{Ib}}$ energetics are within chemical accuracy. To facilitate comparisons, the corresponding FEB-SPQE results are also included.

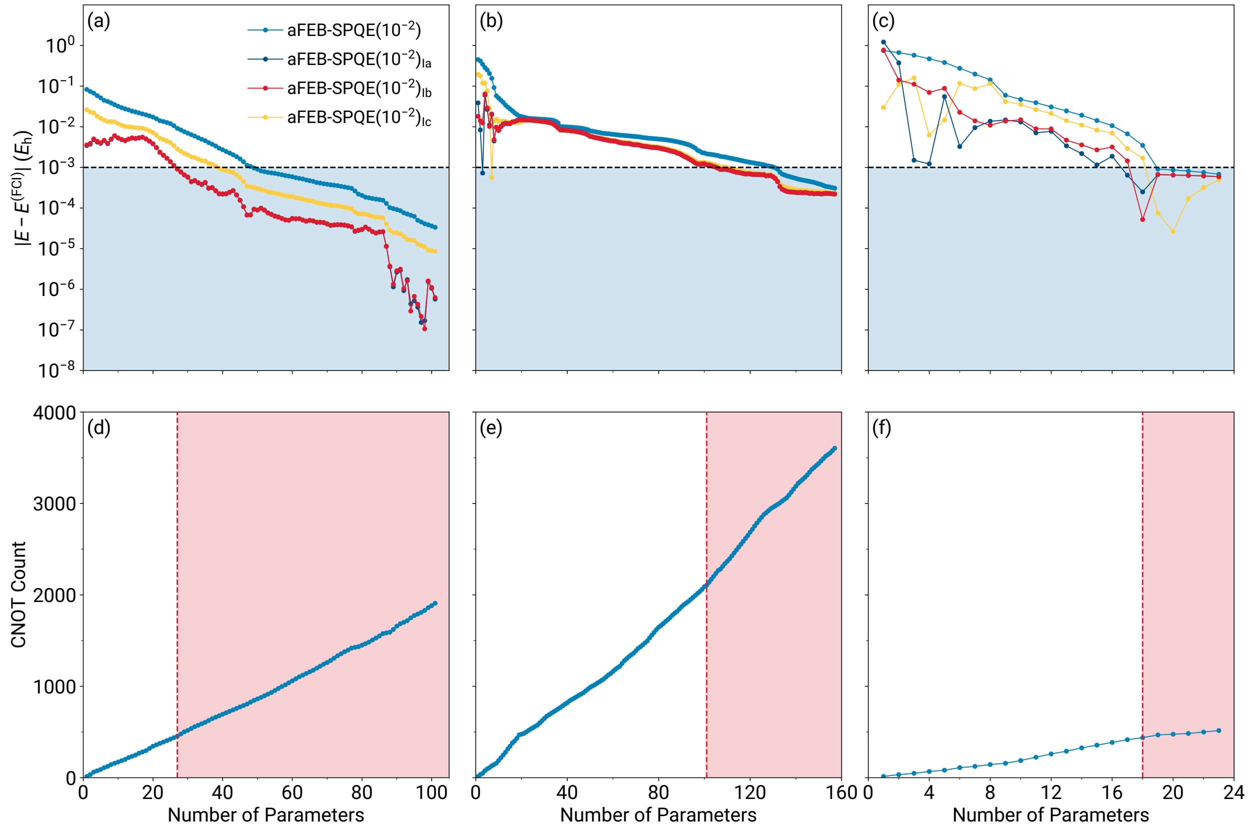

Figure S2: Errors relative to FCI [(a)–(c)] and CNOT gate counts [(d)–(f)] characterizing the aFEB-SPQE simulations of the symmetric dissociation of the H_6/STO-6G linear chain at three representative distances between neighboring H atoms, including $R_{\text{H–H}} = 1.0\,\text{Å}$ [(a) and (d)], $R_{\text{H–H}} = 2.0\,\text{Å}$ [(b) and (e)], and $R_{\text{H–H}} = 3.0\,\text{Å}$ [(c) and (f)]. The blue-shaded area in the top-row panels indicates results within chemical accuracy ($1\,\text{m}E_{\text{h}}$) from FCI. The red-shaded area in the bottom-row panels denotes the CNOT counts of the underlying aFEB-SPQE quantum circuits for which the aFEB-SPQE$_{\text{Ib}}$ energies are within chemical accuracy.

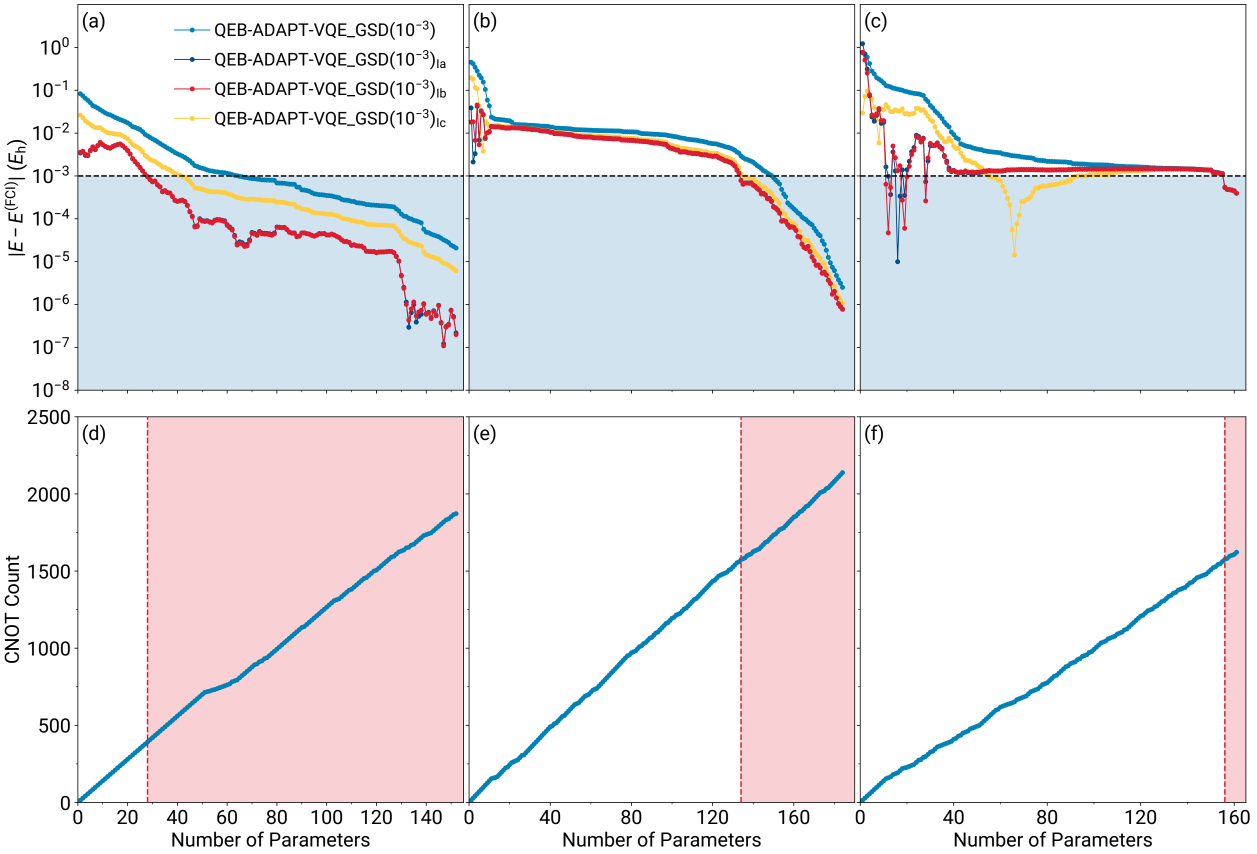

Figure S3: Errors relative to FCI [(a)–(c)] and CNOT gate counts [(d)–(f)] characterizing the QEB-ADAPT-VQE simulations of the symmetric dissociation of the H_6/STO-6G linear chain at three representative distances between neighboring H atoms, including $R_{\text{H–H}} = 1.0\,\text{Å}$ [(a) and (d)], $R_{\text{H–H}} = 2.0\,\text{Å}$ [(b) and (e)], and $R_{\text{H–H}} = 3.0\,\text{Å}$ [(c) and (f)]. The blue-shaded area in the top-row panels indicates results within chemical accuracy ($1\,\text{m}E_{\text{h}}$) from FCI. The red-shaded area in the bottom-row panels denotes the CNOT counts of the underlying QEB-ADAPT-VQE quantum circuits for which the QEB-ADAPT-VQE$_{\text{Ib}}$ energies are within chemical accuracy.
